# Artificial intelligence in gangrenous cholecystitis: advances in risk factor identification and preoperative prediction—a scoping review

**DOI:** 10.3389/fsurg.2026.1934684

**Published:** 2026-09-03

**Authors:** Ping Wang, Xingyu Chen, Tianjiao Hao, Jisong Chen

**Affiliations:** Department of Hepatopancreatobiliary Surgery, Taizhou Fourth People’s Hospital, Taizhou, Jiangsu, China

**Keywords:** artificial intelligence, deep learning, explainable AI, gallbladder gangrene, gangrenous cholecystitis, machine learning, prediction model, scoping review

## Abstract

**Background:**

Gangrenous cholecystitis (GC) is a severe pathological subtype of acute cholecystitis, yet its preoperative diagnostic rate remains below 10%. Conventional scoring systems based on logistic regression demonstrate limited predictive performance. Artificial intelligence (AI) approaches may offer improved risk stratification, but the evidence base remains limited.

**Methods:**

A scoping review was conducted across PubMed, Web of Science, Embase, and CNKI databases for publications from January 2020 to June 2026. The search combined MeSH terms and free-text keywords related to gangrenous cholecystitis and artificial intelligence. After screening 412 records, 18 studies were included: 3 direct GC AI prediction studies, 3 traditional GC scoring systems, and 12 indirect or methodologically related studies. A narrative synthesis was adopted given the substantial heterogeneity in study design.

**Results:**

Three studies directly addressed AI-based GC prediction. Machine learning models using structured clinical data achieved validation AUCs of 0.818–0.944, though these were derived from retrospective, single-center or limited multicenter cohorts. Deep learning models integrating non-contrast and contrast-enhanced CT achieved independent validation AUCs of 0.879 and 0.887 (training-set AUC 0.965). Explainable AI methods identified model-associated predictors, including hypokalemia/hyponatremia, though these require pathophysiological validation. No study reported calibration, decision curve analysis, or prospective clinical impact evaluation.

**Conclusions:**

Early retrospective studies show promising discrimination for AI-based GC prediction; however, evidence remains insufficient for routine clinical decision-making due to methodological heterogeneity, limited external validation, absence of prospective impact studies, and lack of calibration and clinical utility analyses. Prospective multicenter validation and implementation research are needed before clinical adoption.

## 1 Introduction

Acute cholecystitis is among the most common acute abdominal conditions encountered in clinical practice. Epidemiological data from China indicate an annual incidence of approximately 0.15%–0.23% (1). Among its subtypes, gangrenous cholecystitis (GC) is a severe pathological variant, characterized pathologically by full-thickness ischemic necrosis of the gallbladder wall, with a reported prevalence of 2%–30% among acute cholecystitis cases (2, 3). The perioperative complication rate of GC is approximately two- to three-fold higher than that of non-gangrenous cholecystitis, and GC is associated with markedly prolonged hospital stays and increased healthcare expenditures (2, 4). It should be noted that GC represents local gangrenous changes of the gallbladder wall and is distinct from systemic severity (organ dysfunction), which defines Grade III acute cholecystitis according to the Tokyo Guidelines (6).

Preoperative diagnosis of GC has long posed a formidable challenge in hepatobiliary surgery. A retrospective cohort study by El Asmar et al. demonstrated that among 121 patients with histopathologically confirmed GC, only nine (7.4%) were diagnosed preoperatively, with over 90% of GC cases being missed prior to surgery (2). This diagnostic shortfall results in delayed intervention—once GC is confirmed, early surgery is warranted, whereas patients without GC may be managed conservatively or scheduled for elective cholecystectomy (5, 6). Consequently, accurate preoperative identification of patients at high risk for GC is of critical importance for optimizing clinical decision-making and improving patient outcomes.

**Figure 1:**
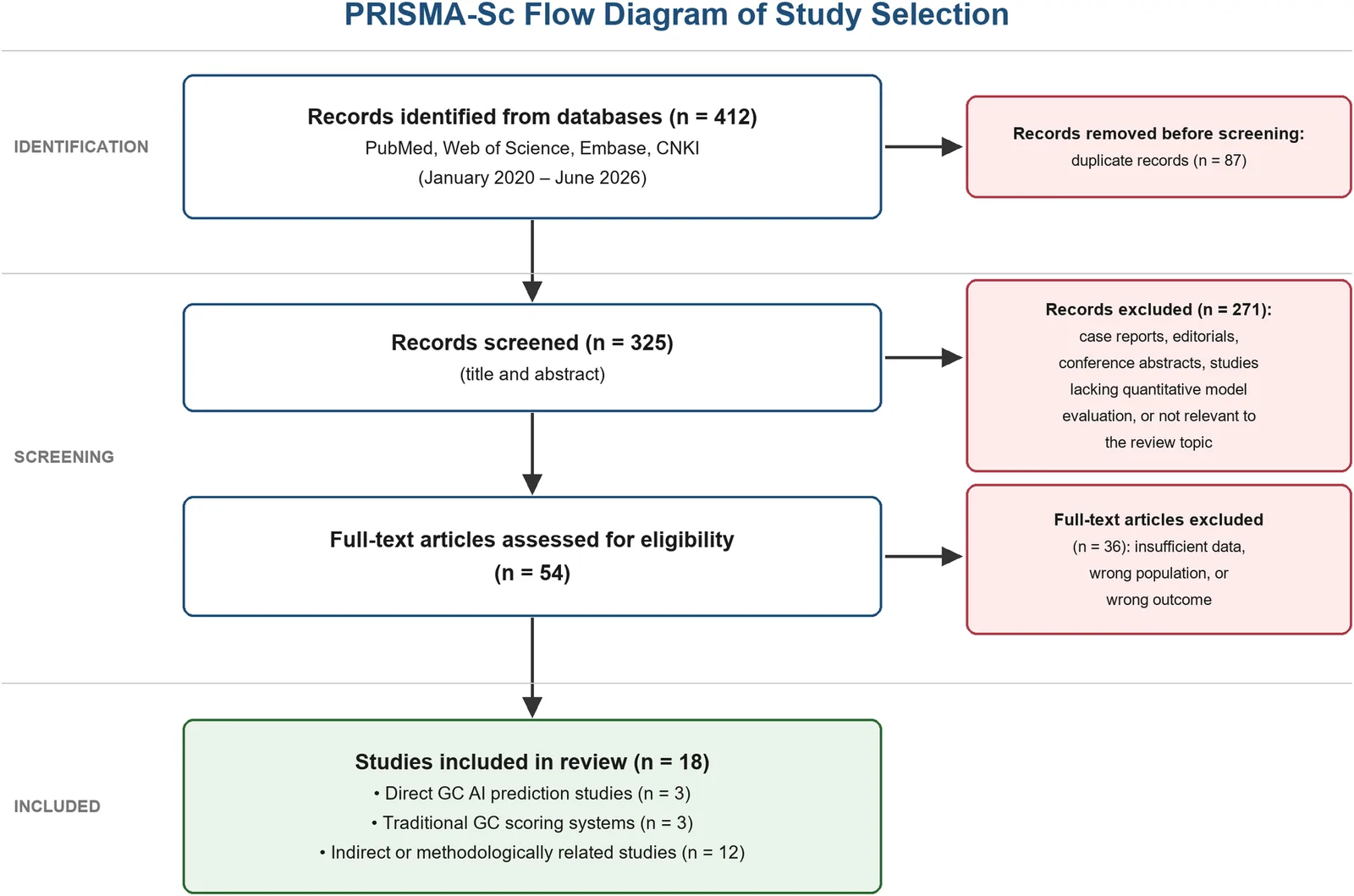
PRISMA-Sc flow diagram of the study selection process. A total of 412 records were identified from PubMed, Web of Science, Embase, and CNKI (January 2020–June 2026). After removal of 87 duplicate records, 325 records were screened by title and abstract, of which 271 were excluded (case reports, editorials, conference abstracts, studies lacking quantitative model evaluation, or not relevant to the review topic). Full-text review of the remaining 54 articles led to exclusion of 36 articles (insufficient data, wrong population, or wrong outcome). Ultimately, 18 studies were included: 3 studies directly addressing AI-based gangrenous cholecystitis prediction or diagnosis, 3 traditional gangrenous cholecystitis scoring systems, and 12 studies providing indirect or methodological evidence from related gallbladder conditions.

The present review employs a scoping review methodology. A comprehensive search was conducted across PubMed, Web of Science, Embase, and the China National Knowledge Infrastructure (CNKI) databases for publications spanning January 2020 to June 2026. The search strategy combined Medical Subject Headings (MeSH) terms with free-text keywords, including “gangrenous cholecystitis,” “gallbladder gangrene,” “artificial intelligence,” “machine learning,” “deep learning,” and “prediction model,” connected by Boolean operators (AND/OR). The initial search yielded 412 records. After removal of 87 duplicates, 325 records were screened by title and abstract, of which 271 were excluded (case reports, editorials, conference abstracts, studies lacking quantitative model evaluation, or studies not relevant to the review topic). Full-text review of the remaining 54 articles led to exclusion of 36 articles (insufficient data, wrong population, or wrong outcome). Ultimately, 18 studies were included: 3 studies directly addressing AI-based GC prediction or diagnosis (17–19), 3 traditional GC scoring systems (9, 33, 34), and 12 studies providing indirect or methodological evidence from related gallbladder conditions (20–22, 25–27, 37–42) (Table 1). Data extraction was performed by one reviewer and verified by a second reviewer; disagreements were resolved through consensus discussion. Given the considerable heterogeneity in study design, algorithm type, and outcome measures among the included studies, a narrative synthesis—rather than a quantitative meta-analysis—was adopted. This review is reported in accordance with the PRISMA-Sc (Preferred Reporting Items for Systematic Reviews and Meta-Analyses extension for Scoping Reviews) guidelines; a PRISMA-style flow diagram of the study selection process is provided in Figure 1.

**Table 1:** Summary of direct GC AI prediction studies and related models.

Study	Year	Population/condition	Algorithm	Cohort (N/GC)	Validation type	AUC	Sensitivity	Specificity	Key notes
Ma et al. (17)	2025	GC (direct)	XGBoost	897 dev/208 GC; 109 temporal test	Temporal holdout (single-center)	0.944 (temporal test)	Not reported	Not reported	7-variable simplified model; online app; SHAP. No calibration/DCA reported
Hu et al. (18)	2025	GC (direct)	Random Forest	1,044/210 GC	Multicenter external validation	0.818 (external val)	Not reported	Not reported	LASSO + Boruta; 10 predictors; SHAP. No calibration/DCA reported
Guo et al. (19)	2025	GC (direct)	seResNet-50 (SSL)	1,228/not reported	Independent validation (2 validation centers of 3 total)	0.965 (training); 0.879/0.887 (validation)	0.88 (train); 0.77/0.76 (val)	0.95 (train); 0.83/0.84 (val)	CT fusion (non-contrast + contrast); outperformed radiologists (AUC 0.721/0.703). Training AUC should not be conflated with validation
Bouassida et al. (9)	2021	GC (traditional score)	Logistic regression	Not specified	Development cohort	0.83	Not reported	Not reported	6-factor score; no imaging integration; no external validation
Wu et al. (33)	2014	GC (traditional score)	Logistic regression	5,243/not specified	Development cohort	0.77	Not reported	Not reported	4-factor simplified score; no external validation
Yacoub et al. (34)	2010	GC (traditional score)	Logistic regression	Not specified	Development cohort	0.75–0.78	Not reported	Not reported	5-factor score; no external validation
Chen et al. (41)	2026	Acute suppurative cholecystitis (indirect)	Stacking ensemble + radiomics	Multicenter/not specified	External test set	0.82 (external test)	0.714 (external test)	0.831 (external test)	Clinical-radiomics fusion; SHAP. ASC, not GC
Jayanthi et al. (39)	2026	Gallbladder diseases (indirect)	SE-CapsNet + BiLSTM	10,692 images	Test set	Not reported (GC-specific)	0.976 (C4 class)	0.993 (C4 class)	Ultrasound DL; 9-class; GC as C4. No GC-specific AUC
Das et al. (38)	2026	Gallstone disease (indirect)	CatBoost (SLOA)	319 patients	5-fold cross-validation	0.82–0.92	Not reported	Not reported	Tabular data, not ultrasound images; gallstone disease, not GC
Cicerone et al. (37)	2025	Cholecystitis complications (indirect)	Machine learning	Not specified	Development + validation	0.846/0.890	Not reported	Not reported	CholeSurgRisk I/II; complications, not GC prediction

Training-set performance is shown for reference only and should not be conflated with validation performance. Direct GC studies are listed first. Indirect evidence studies are listed separately and should not be used to draw conclusions about GC prediction performance. GC, gangrenous cholecystitis; AUC, area under the receiver operating characteristic curve; SSL, self-supervised learning; DCA, decision curve analysis; dev, development cohort; val, validation.

Traditional identification of GC risk factors has relied predominantly on univariate analysis and multivariable logistic regression. Currently established risk factors include advanced age, male sex, diabetes mellitus, cardiovascular disease, elevated white blood cell (WBC) count, elevated C-reactive protein (CRP), gallbladder distension, and pericholecystic fluid (2, 7, 8). Clinical scoring systems constructed from these risk factors, such as the Yacoub score, have been applied but demonstrate only modest predictive performance (AUC 0.75–0.78) (9). These scoring tools harbor several inherent limitations—they assume linear relationships among variables, incorporate a limited number of predictors, and largely fail to integrate imaging information (10).

In recent years, artificial intelligence (AI) technologies have advanced rapidly in medicine (11, 12). Machine learning (ML) algorithms can discern non-linear relationships within high-dimensional data (13, 14), deep learning (DL) models can automatically extract features from imaging data (15), and explainable AI (XAI) provides methodological support for bridging the gap between “black-box” models and clinical trust (16). In the GC domain, three studies have directly applied AI to GC prediction: Ma et al. (17) achieved a temporal validation AUC of 0.944 using XGBoost on a single-center development cohort of 897 patients (with a temporal holdout of 109 patients); Hu et al. (18) developed a multicenter random forest model with an external validation AUC of 0.818; and Guo et al. (19) integrated self-supervised learning with multicenter CT imaging, achieving independent validation AUCs of 0.879 and 0.887 (training-set AUC 0.965). These preliminary studies suggest that AI-based approaches may offer improved GC risk stratification, though the evidence base remains limited and requires cautious interpretation.

The present review aims to examine the application of AI technologies in GC risk factor identification and prediction over the past five years, spanning three dimensions: machine learning models for structured clinical data, deep learning models for imaging data, and emerging AI technologies. We further discuss current challenges, methodological limitations, and future directions, with the goal of providing a reference framework for precision diagnosis and treatment of GC. Importantly, conclusions regarding GC prediction performance are based primarily on the three direct GC studies; evidence from related conditions is discussed separately as indirect or methodological reference.

## 2 Traditional risk factors for gangrenous cholecystitis

### 2.1 Demographic and comorbidity factors

Traditional risk factors for GC have been extensively investigated. With respect to demographics, advanced age is the most consistently identified independent risk factor. El Asmar et al. (2) reported in a retrospective cohort study that, after adjusting for sex, diabetes, coronary heart disease, and hypertension, each one-year increase in age was associated with an approximately 3% increase in the risk of acute gangrenous cholecystitis (OR = 1.03, 95% CI: 1.01–1.05, *p* = 0.009). Liu et al. (20) similarly confirmed that age ≥60 years constitutes an independent risk factor. Male sex represents another independent predictor. Multivariable logistic regression analysis by El Asmar et al. (2) showed that the risk of developing acute gangrenous cholecystitis in males was approximately 1.9 times that in females (OR = 1.897, 95% CI: 1.024–3.513, *p* = 0.042). Notably, some heterogeneity persists across studies regarding whether male sex qualifies as an independent predictor; certain investigations failed to retain male sex as an independent risk factor in multivariable analysis (21), a finding potentially attributable to differences in population characteristics, sample size, and collinearity—warranting further exploration.

With respect to comorbidities, diabetes mellitus (DM) is the most extensively studied comorbidity-related risk factor, with multiple studies confirming that DM patients face a significantly elevated risk of progression from acute cholecystitis to gangrene (2, 22). El Asmar et al. (2) identified DM as an independent predictor of GC complicated by sepsis (OR = 2.67, 95% CI: 1.08–6.60, *p* = 0.033). Cardiovascular disease constitutes an important risk factor for GC, with its mechanism primarily mediated through gallbladder wall hypoperfusion secondary to atherosclerotic changes in the cystic artery (23). Underlying cardiovascular pathology—such as coronary atherosclerosis and heart failure—can intrinsically cause thickening of the gallbladder microvasculature and luminal narrowing. In the setting of superimposed systemic hypoperfusion events (e.g., acute myocardial infarction, open-heart surgery, aortic surgery), cystic artery perfusion is further compromised, leading to gallbladder wall (particularly mucosal layer) ischemia and necrosis, with rapid progression to gangrenous cholecystitis (23). Furthermore, vagal reflexes triggered by cholecystitis may exacerbate myocardial ischemia, establishing a vicious “ischemia-inflammation” cycle that further elevates the risk of GC (23). Portal hypertension-induced gallbladder wall edema, congestion, and coagulopathy also jointly contribute to increased GC risk and surgical complexity (24).

### 2.2 Laboratory parameters

With respect to laboratory parameters, elevated WBC count is the most classical biomarker for GC. According to the Tokyo Guidelines 2018 (TG18), WBC >18,000/mm^3^ and marked local inflammation, including gangrenous cholecystitis, are classified as Grade II (moderate) acute cholecystitis criteria; Grade III (severe) acute cholecystitis is defined by organ dysfunction (6). Procalcitonin (PCT) exhibits substantially higher specificity for cholecystitis complicated by severe systemic infection compared with CRP (92.1% vs. 69.7%), achieving a negative predictive value of 96.5% at a cutoff of ≥5.095 ng/mL (25). Elevated D-dimer (>1.18 μg/mL) is an independent risk factor for gallbladder gangrene (OR = 4.03), implicating local circulatory disturbance and gallbladder wall microthrombosis in the pathogenesis of GC (21). The neutrophil-to-lymphocyte ratio (NLR) and the Systemic Immune-Inflammation Index (SII) are composite inflammatory indices that have attracted considerable attention in recent years. Zheng et al. (21) demonstrated that SII significantly outperformed CRP, WBC, and PCT in predicting GC (AUC: 0.880 vs. 0.797/0.726/0.773, *p* < 0.05), a finding subsequently corroborated by Feier et al. (26) (SII AUC = 0.889). It should be noted that the Zheng et al. study specifically enrolled patients with acute acalculous cholecystitis, and these findings should not be generalized to all patients with calculous acute cholecystitis without further validation. Non-neoplastic elevation of CA19-9 in GC is a phenomenon increasingly recognized in recent years. Zheng et al. (21) identified CA19-9>18.8 U/mL as an independent risk factor for gallbladder gangrene (OR = 7.21), with the putative mechanism involving severe inflammation-induced damage to biliary epithelial cells and increased CA19-9 release (21, 27). Notably, thrombocytosis during the active phase of GC (platelet count >351 × 10^9^/L) reflects a systemic inflammation-driven hypercoagulable state and serves as an independent risk factor for gallbladder gangrene (21); conversely, when GC is complicated by sepsis or disseminated intravascular coagulation (DIC), platelet counts may decline markedly due to consumptive coagulopathy (28).

### 2.3 Imaging features

With respect to imaging, gallbladder distension is a strong predictor of GC. El Asmar et al. (2) determined, through ROC curve analysis, that a transverse gallbladder diameter >3.85 cm represents the optimal cutoff for differentiating GC from non-gangrenous cholecystitis. Multivariable logistic regression demonstrated that patients with a transverse diameter >3.85 cm faced an approximately 3.42-fold increased risk of GC compared with those with a diameter ≤3.85 cm (OR = 3.42, 95% CI: 1.786–6.562, *p* < 0.001). Zheng et al. (21) likewise confirmed that gallbladder distension is an independent risk factor for gallbladder gangrene (multivariable OR = 6.47, 95% CI: 2.19–19.10, *p* < 0.001), with a detection rate of 63.7% in the gangrene group, substantially higher than the 18.5% observed in the non-gangrene group.

Gallbladder wall thickening (≥4 mm) is a classical diagnostic criterion for acute cholecystitis (6); however, it should be noted that this sign *per se* is not specific for GC—it can occur in both simple and gangrenous cholecystitis, reflecting inflammatory edema of the gallbladder wall (29). The key imaging findings truly indicative of GC are structural loss within necrotic regions of the gallbladder wall, manifested on ultrasound as loss of the mucosal line/wall interface echogenicity, wall de-lamination, asymmetric wall thickening, or intraluminal floating necrotic membranes, with an incidence exceeding 90% (29, 30). Color Doppler ultrasound (CDUS) and contrast-enhanced ultrasound (CEUS) further enhance the specificity of GC diagnosis by demonstrating focal perfusion defects in the gallbladder wall—CEUS-demonstrated wall perfusion defects can achieve a specificity of 100% (PPV 100%) (31). On contrast-enhanced CT, GC manifests as heterogeneous wall enhancement, focal non-enhancing areas, or even wall discontinuity (irregular or absent wall) (32); in a study of 31 GC cases, focal reduced enhancement was observed in 80% of patients undergoing contrast-enhanced scanning (30). In essence, wall thickening reflects the degree of inflammation, whereas wall thinning/structural loss/perfusion defects represent the specific signs indicative of gangrenous transformation (6, 31, 32).

### 2.4 Limitations of traditional scoring systems

Several GC prediction scoring systems have been developed based on the aforementioned factors. Wu et al. (33), based on a retrospective analysis of 5,243 cholecystectomy patients, proposed a simplified scoring system (0–5 points) comprising four factors: age (stratified scoring: ≤45 years = 0, 46–65 years = 1, >65 years = 2), heart rate >90 bpm, WBC >13,000/mm^3^, and gallbladder wall thickening ≥4 mm, achieving a modified AUC of 0.77. Earlier, Yacoub et al. (34) proposed a five-factor score (0–6.5 points) including male sex (2 points), age >45 years (1 point), heart rate >90 bpm (1 point), WBC >13,000/mm^3^ (1.5 points), and gallbladder wall thickness >4.5 mm (1 point); however, male sex lost statistical significance in Wu et al.'s multivariable analysis. Bouassida et al. (9) further proposed a new scoring system in 2021 incorporating six factors—ASA score 3–4, body temperature, symptom duration, WBC, male sex, and pericholecystic fluid—achieving an AUC of 0.83, outperforming both prior scoring systems.

Nevertheless, these scoring systems harbor inherent limitations: (1) reliance on the linearity assumption of logistic regression, rendering them incapable of capturing interaction effects and non-linear relationships among variables; (2) incorporation of a limited number of variables, resulting in suboptimal information utilization; (3) lack of deep integration of imaging features; (4) absence of external and prospective validation in the majority of cases; (5) inability to provide individualized contribution analysis, yielding insufficient clinical interpretability; (6) absence of a temporal dimension—all scores are derived from single cross-sectional data at admission; (7) failure to integrate novel composite inflammatory biomarkers, such as NLR, SII, and D-dimer; and (8) incomplete reporting of model calibration and decision curve analysis (DCA) for published scoring systems, hindering assessment of their net clinical benefit in real-world settings. These limitations collectively provide methodological rationale for exploring AI technologies in GC prediction.

## 3 Machine learning prediction models based on structured data (direct GC evidence)

### 3.1 Application of traditional machine learning algorithms

In 2025, Ma et al. (17) published the largest GC machine learning prediction study to date. This single-center investigation enrolled 897 patients in the development cohort (208 in the GC group and 689 in the non-GC group) and employed a temporal holdout of 109 patients as the test set. The authors collected 26 clinical features and constructed and compared five models: decision tree, support vector machine (SVM), random forest (RF), XGBoost, and AdaBoost. Through five-fold cross-validation, XGBoost was identified as the optimal model, achieving an AUC of 0.944 (95% CI: 0.888–0.984) on the temporal test set (*n* = 109). It should be noted that this was a temporal rather than geographical external validation, as the test cohort was drawn from the same institution. The study also developed a streamlined model comprising only seven variables (WBC, NLR, D-dimer, fibrinogen, gallbladder width, gallbladder wall thickness, and hypokalemia/hyponatremia), which maintained high predictive performance while reducing data acquisition costs, and released a Streamlit-based online prediction application (https://gangrenous-cholecystitis-prediction-model.streamlit.app/), enabling real-time probability prediction and visualization of variable contributions. Of note, the AUC of 0.944 and a reported preoperative clinical diagnostic accuracy of 86.95% are not directly comparable, as they represent different metrics (discrimination vs. accuracy) assessed under different conditions; no formal paired statistical comparison was performed.

In the same year, Hu et al. (18) published the first multicenter GC machine learning prediction model. Employing multicenter retrospective data, the study enrolled 1,044 patients (521 in the training set, 223 in the test set, 300 in the external validation set), including 210 GC patients. Through dual variable selection using LASSO regression and the Boruta algorithm, 10 predictors were ultimately identified, and a random forest-based prediction model was developed and externally validated, achieving an AUC of 0.818 (95% CI: 0.763–0.873) on the external validation set. SHAP (SHapley Additive exPlanations) analysis identified gallbladder wall thickness, CRP, pericholecystic fluid, WBC, and impacted stones as the five most important predictive features. Multicenter external validation provided preliminary evidence of the model's generalizability, though further prospective validation is needed.

These two studies represent the current state of the art in structured-data ML prediction for GC. Notably, Ma et al. (17) found that commonly used oversampling methods such as SMOTE paradoxically exacerbated overfitting in GC prediction—a finding of methodological significance for future research. A marked difference in AUC exists between the two studies (0.944 vs. 0.818); however, no conclusion about the superiority of a specific algorithm can be drawn from cross-study AUC values, as differences may reflect case mix, eligibility criteria, GC prevalence, predictor availability, outcome ascertainment, and validation design rather than algorithmic superiority. Ma et al. used single-center data with temporal validation, which may have led to overfitting of center-specific patterns, whereas Hu et al. provided more conservative performance estimates through multicenter external validation. Additionally, Ma et al.'s model incorporated hypokalemia/hyponatremia, a highly discriminative variable (GC group 87.7% vs. non-GC group 48.1%), which may have substantially enhanced discriminative capacity. Future studies should conduct head-to-head model benchmarking under a unified variable set and standardized preprocessing pipeline to more reliably assess the relative merits of different algorithms in GC prediction.

### 3.2 Introduction of explainable artificial intelligence

Although machine learning models have demonstrated promising performance in GC prediction, their “black-box” decision-making mechanisms impede clinical adoption. Surgeons need to understand why a model makes a given prediction before incorporating it into clinical decision-making. Explainable AI (XAI) serves as a bridge across this chasm by providing transparent explanations of model decisions (35). Mainstream XAI methods such as SHAP quantify the contribution of each clinical feature to the prediction outcome, thereby visualizing the model's decision-making process and facilitating clinicians' understanding of model predictions.

In the study by Ma et al. (17), SHAP analysis identified hypokalemia/hyponatremia as an important model-associated predictor of GC (present in 87.7% of the GC group vs. 48.1% of the non-GC group). It is important to emphasize that SHAP quantifies how features contribute to predictions made by a specific fitted model; it does not identify novel causal risk factors. Hypokalemia/hyponatremia should therefore be described as a model-associated predictor rather than a novel risk factor “discovered” by AI. The pathophysiological significance of this association requires further investigation, as it may reflect confounding by disease severity, treatment effects, centre-specific measurement practices, or the timing of laboratory measurements relative to disease onset. In Hu et al.'s (18) multicenter model, SHAP ranking identified gallbladder wall thickness as the most important predictive feature, surpassing traditional markers such as WBC and CRP—a finding consistent with the pathophysiological mechanism of ischemic necrosis of the gallbladder wall, though again, SHAP importance should not be interpreted as proof of pathophysiological relevance or causality.

The value of SHAP extends beyond feature importance ranking to its ability to explain each individual prediction. Ma et al.'s (17) online application not only provides a predicted probability but also displays the positive or negative contribution of each variable to the prediction, enabling surgeons to assess whether the model's prediction aligns with clinical intuition. This individualized explanation is a capability that traditional scoring systems cannot offer. However, several important limitations of SHAP-based explanations should be acknowledged: (1) SHAP explanations are model-specific—a feature deemed important by one model may not be important in another; (2) SHAP importance does not imply causal relevance, as correlations may be confounded; (3) explainability methods can be unstable, with small changes in data or model parameters potentially leading to different feature rankings; and (4) whether clinicians truly benefit from these explanations during real-world decision-making requires empirical evaluation, as the relationship between explanation provision and clinical decision quality is not straightforward.

Beyond SHAP, LIME (Local Interpretable Model-agnostic Explanations) represents another important model-agnostic XAI method, which explains individual predictions by fitting a locally linear approximation model in the neighborhood of the sample to be explained (36). However, as of 2026, no published report on the application of LIME in GC prediction exists. Future research may explore the combined use of SHAP and LIME—employing SHAP for global feature importance ranking and LIME to assist with fine-grained explanations for specific high-risk patients—to further enhance the clinical interpretability of GC prediction models.

### 3.3 Development of online prediction tools

The clinical value of AI prediction models ultimately hinges on their accessibility and usability in real-world clinical settings. In recent years, online prediction tools for GC have begun to emerge:

Ma et al.'s (17) Streamlit-based online application is currently the most comprehensive GC prediction tool, generating a GC probability and visualizing the contribution of each variable upon entry of seven clinical parameters.

Hu et al. (18) designed a clinical decision-support tool based on their multicenter model, aimed at assisting surgeons in rapidly assessing GC risk.

In the broader domain of biliary surgery, Cicerone et al. (37) developed two machine learning-based risk prediction tools in 2025—CholeSurgRisk I (preoperative model, AUC = 0.846) and CholeSurgRisk II (comprehensive model, AUC = 0.890)—for predicting the risk of major complications following laparoscopic cholecystectomy for acute calculous cholecystitis. The dissemination of the preoperative online tool also provides a reference paradigm for the development of GC prediction models.

The emergence of these online tools signals that AI-based GC prediction is transitioning from academic research toward clinical practice; however, their real-world effectiveness and clinical impact remain unknown and require evaluation through prospective studies. It should be emphasized that the availability of an online tool does not constitute evidence of clinical utility.

## 4 Deep learning models based on imaging data

### 4.1 CT imaging deep learning (direct GC evidence)

CT is the most commonly employed imaging modality for preoperative GC evaluation. In 2025, Guo et al. (19) published the first multicenter GC imaging deep learning study to date. This investigation enrolled 1,228 patients from three centers, yielding 7,368 CT images (training set, 921 patients; independent validation set I, 143 patients; validation set II, 164 patients). A self-supervised learning (SSL) strategy was implemented using the seResNet-50 framework, integrating both non-contrast and contrast-enhanced CT modalities. The SSL strategy involved pre-training on large volumes of unlabeled CT images, followed by fine-tuning on limited labeled data, thereby effectively mitigating the bottleneck of scarce medical image annotations. The results demonstrated that the fusion model achieved an AUC of 0.965 on the training set and 0.879 and 0.887 on independent validation sets I and II, respectively—significantly outperforming the contrast-enhanced CT unimodal model (0.791/0.810) and the non-contrast CT unimodal model (0.756/0.730) (DeLong test, *P* < 0.001). The training set sensitivity and specificity were 88% and 95%, respectively; validation set I sensitivity and specificity were 77% and 83%; and validation set II sensitivity and specificity were 76% and 84%. Notably, the diagnostic performance of the fusion model in validation set I (AUC 0.879) surpassed that of two senior radiologists (AUC 0.721 and 0.703, respectively). It is important to emphasize that the AUC of 0.965 refers to training-set performance; the independent validation AUCs of 0.879 and 0.887 represent the more clinically relevant metrics for assessing generalizability.

This study demonstrates that CT imaging deep learning shows promise for GC diagnosis; nevertheless, AI and radiological interpretation remain complementary—AI can detect subtle imaging features that may be imperceptible to the human eye, whereas the macroscopic judgment and clinical experience of radiologists remain indispensable.

### 4.2 Ultrasound image AI analysis (indirect evidence)

Ultrasound is the first-line imaging modality for acute cholecystitis; however, AI analysis of ultrasound images poses considerably greater challenges than CT, owing to the operator-dependent nature of ultrasound image quality and low standardization. Despite these challenges, preliminary progress has been achieved in this domain, though no study has specifically developed an ultrasound-based AI model for GC.

Das et al. (38) utilized clinical tabular data from 319 patients with gallstone disease (38 features) sourced from the UCI database to develop a predictive model for gallstone disease onset based on the CatBoost classifier and the Sea Lion Optimization Algorithm (SLOA). This study used tabular clinical data rather than ultrasound images and targeted gallstone disease rather than GC; its relevance to GC prediction is therefore indirect and methodological. The SLOA simultaneously performed dual tasks—selecting 19 features from the original 38 and optimizing CatBoost hyperparameters. Through five-fold cross-validation, the SLOA_CB model achieved an accuracy of 80.42% (AUC 0.82–0.92). The study also employed three XAI methods—SHAP, LIME, and DiCE—to construct a multi-layered interpretability framework. Although this study does not constitute ultrasound-image AI for GC, its combined optimization and multi-method XAI framework provides methodological reference for clinical data modeling.

A study that addressed the ultrasound imaging level was that of Jayanthi et al. (39), which constructed a hybrid deep learning model—HDLMFE-ADGDT—covering nine gallbladder diseases based on 10,692 ultrasound images. The model employed SE-CapsNet for feature extraction combined with CNN-BiLSTM for sequence classification, achieving an overall accuracy of 99.09% (sensitivity 95.87%, specificity 99.49%). Of particular relevance to GC, this study designated category 4 (C4) as an exclusive classification for membranous and gangrenous cholecystitis, attaining a test-set sensitivity of 97.64% and specificity of 99.31%. However, this study was not specifically designed for GC prediction and did not report GC-specific AUC, calibration, or clinical utility metrics. In comparison with classical architectures such as VGG16 (97.78%), ResNet152 (85.30%), and InceptionV3 (86.50%), the SE-CapsNet plus BiLSTM hybrid approach demonstrated clear superiority in ultrasound image classification of gallbladder diseases.

Given that ultrasound is the most commonly used initial imaging examination for acute cholecystitis, the development of GC-specific ultrasound AI models holds significant clinical value and broad application prospects. However, this remains an unmet need, as no published study has directly addressed this question.

### 4.3 Radiomics (indirect evidence)

The radiomics paradigm involves extracting high-throughput quantitative features from medical images and converting this imaging information into mineable data. Gupta et al. (40) conducted an early exploration of CT texture analysis in gallbladder cancer, employing TexRAD software to process portal venous phase CT images from 38 patients with suspected gallbladder cancer. This study concerns gallbladder cancer rather than GC and is therefore discussed here as methodological reference. Filter-histogram-based texture analysis identified consistent texture parameters—mean intensity and kurtosis—as predictors of adenocarcinoma histological grade, with kurtosis significantly associated with overall survival (*p* = 0.020). Its methodological framework—comprising ROI delineation, multi-scale texture feature extraction, and association analysis between texture parameters and clinical outcomes—provides a transferable technical pathway for subsequent radiomics-based investigation of GC.

Chen et al. (41) integrated clinical features with CT radiomic features in 2026, employing a stacking ensemble strategy to construct an explainable machine learning model for preoperative prediction of acute suppurative cholecystitis (ASC). This study targeted ASC rather than GC and is discussed as indirect evidence. In this multicenter retrospective study, a clinical model (AUC = 0.75), a radiomics model (AUC = 0.76), and a clinical-radiomics fusion model (AUC = 0.82) were separately constructed, with the fusion model achieving a sensitivity of 71.4% and specificity of 83.1% on the external test set. The model also provided feature-level explanations through SHAP analysis. This study offers methodological reference for predicting cholecystitis severity from a clinical-radiomics fusion perspective.

### 4.4 Multimodal fusion strategies

Multimodal fusion represents a frontier direction in current medical AI. Guo et al.'s (19) study confirmed that the fusion of non-contrast and contrast-enhanced CT significantly outperforms either modality alone in independent validation. The underlying mechanism resides in the complementary imaging information provided by the two modalities—non-contrast CT reflects baseline tissue density characteristics, whereas contrast-enhanced CT reveals perfusion status. Given that the core pathology of GC is ischemic necrosis, non-enhancing regions of the gallbladder wall on contrast-enhanced CT carry direct diagnostic significance for GC.

Looking forward, more advanced multimodal fusion strategies warrant exploration: combined clinical data plus imaging feature models—fusing ML-extracted clinical risk factors with DL-extracted imaging features at the feature or decision level holds promise for constructing a more comprehensive GC prediction framework; ultrasound plus CT plus clinical parameter trimodal fusion—with ultrasound providing real-time bedside assessment, CT delivering in-depth imaging information, and clinical parameters offering systemic status evaluation. However, these strategies remain largely hypothetical and require empirical validation.

## 5 Emerging AI technologies: future research perspectives

The following sections discuss emerging AI technologies that have been proposed as potential future directions for GC research. It should be emphasized that these technologies are largely speculative in the GC context, with little or no GC-specific evidence currently available. They are presented as future research perspectives rather than established approaches.

### 5.1 Potential roles of large language models

Large language models (LLMs) are transforming medical information processing. In the GC domain, potential LLM applications include: (1) electronic health record text mining—natural language processing (NLP) techniques may parse unstructured electronic health records to extract supplementary risk factor information; (2) clinical decision support—LLMs may integrate clinical practice guidelines with individualized risk assessments, though they carry the risk of generating false or misleading medical information (“hallucination”); and (3) patient education—LLMs may generate educational materials and preoperative discussion talking points. These applications remain hypothetical in the GC context and have not been validated.

### 5.2 Federated learning and multicenter collaboration

The generalizability of GC-AI models requires diverse training data; however, medical data privacy protection imposes constraints on cross-institutional data sharing. Federated learning offers a potential solution by enabling each healthcare institution to train models locally, sharing only model parameters rather than raw data. Hu et al.'s (18) multicenter model employed a conventional centralized data integration strategy. The future incorporation of a federated learning framework may allow inclusion of heterogeneous data from more centers while preserving data privacy, though this has not been implemented in GC research to date.

### 5.3 Surgical video AI analysis (indirect evidence)

In 2022, Ward et al. (42) developed an AI model based on laparoscopic cholecystectomy videos capable of automatically identifying the Parkland Grading Scale (PGS) of gallbladder inflammation. This study employed deep learning to analyze surgical video frames and predicted operative duration (R^2^ = 0.61) and the attainment of the Critical View of Safety (CVS). It should be noted that the AI model was designed to identify the PGS rather than to directly predict GC; furthermore, inflammation severity had no substantial effect on CVS attainment and only a limited average association with gallbladder injury. This study provides methodological reference for intraoperative AI applications but does not constitute direct evidence for GC prediction.

Although no published studies have reported intraoperative real-time AI identification of GC, the feasibility of video-based AI analysis has been validated in related contexts. Intraoperative recognition of GC wall necrosis features could potentially assist surgeons in adjusting surgical strategy; however, this remains speculative.

### 5.4 Temporal data analysis

All current GC prediction models are static, cross-sectional predictors—rendering judgments based on single-time-point data at admission. GC development is a dynamic process, and temporal models such as recurrent neural networks (RNNs) and long short-term memory networks (LSTMs) may potentially track the temporal evolution of inflammatory markers. However, no published study has applied temporal modeling to GC prediction.

## 6 Critical appraisal of included prediction studies

High discrimination alone does not establish clinical readiness. A formal critical appraisal of the three direct GC prediction studies (17–19) was conducted using domains consistent with PROBAST + AI and TRIPOD + AI guidelines (Table 2). The following key methodological concerns were identified:

**Table 2:** Critical appraisal of direct GC AI prediction studies using PROBAST + AI and TRIPOD + AI domains.

Appraisal domain	Ma et al. (17)	Hu et al. (18)	Guo et al. (19)
Study design	Retrospective, single-center	Retrospective, multicenter	Retrospective, multicenter
Selection bias	Surgery-only cohort; partial verification bias	Surgery-only cohort; partial verification bias	Surgery-only cohort; partial verification bias
GC event count	208 (development); temporal test not specified	210	Not reported
Class imbalance	Addressed; SMOTE found harmful	Not reported	Not reported
Validation type	Temporal holdout (same center)	Multicenter external validation	Independent validation (2 centers)
Geographical validation	No (single center)	Yes (multicenter)	Yes (2 independent centers)
Data splitting	Patient-level	Patient-level	Not fully described (image-level risk)
Missing-data handling	Not reported	Not reported	Not reported
Feature selection leakage	Not described in detail	LASSO + Boruta (dual selection)	SSL pre-training; details limited
Calibration	Not reported	Not reported	Not reported
Decision curve analysis	Not reported	Not reported	Not reported
PPV/NPV	Not reported	Not reported	Not reported
Sensitivity/Specificity	Not reported	Not reported	Reported (training and validation)
Confidence intervals	Reported for AUC	Reported for AUC	Not reported for AUC
Code availability	Not available (online app only)	Not available	Not available
PROBAST + AI overall risk	High (single-center, temporal only)	Moderate (multicenter, but limited reporting)	Moderate (multicenter, but incomplete reporting)

PROBAST + AI, Prediction model Risk Of Bias ASsessment Tool, adapted for AI; TRIPOD + AI, Transparent Reporting of a multivariable prediction model for Individual Prognosis Or Diagnosis, adapted for AI. “High” risk indicates significant concerns about the reliability of performance estimates; “Moderate” indicates some concerns but not disqualifying. All three studies would benefit from prospective validation and more complete reporting.

Selection bias and retrospective design: All three studies employed retrospective designs. Ma et al. (17) and Hu et al. (18) included only patients who underwent cholecystectomy, meaning that the training labels were derived exclusively from surgical patients; unrecognized GC may exist among patients who improved with conservative management, introducing partial-verification bias. Guo et al. (19) similarly relied on postoperative pathological confirmation.

Sample size and class imbalance: The number of GC events was relatively small (208, 210, and unspecified across studies), limiting the statistical power for detecting model performance differences and increasing the risk of overfitting, particularly for deep learning models with millions of parameters.

Validation strategy: Ma et al. (17) employed temporal holdout validation within a single center, which does not address geographical generalizability. Hu et al. (18) provided multicenter external validation, offering stronger evidence of generalizability. Guo et al. (19) reported independent validation from two centers, though details on patient-level vs. image-level splitting were not fully described.

Calibration and clinical utility: None of the three studies reported calibration curves, decision curve analysis, positive or negative predictive values, or net benefit analyses. Without these metrics, the clinical usefulness of the models cannot be adequately assessed, as high discrimination (AUC) alone does not guarantee clinical utility.

Reproducibility: None of the studies made their code or models publicly available in a reproducible format (with the exception of Ma et al.'s online Streamlit application, which does not provide access to the underlying model). This limits independent verification and external replication.

Predictor timing and availability: Several predictors used in the models (e.g., intraoperative findings, specific laboratory values) may not be available at the intended clinical decision point (e.g., emergency department triage), potentially limiting real-world applicability.

Missing-data handling: The studies did not consistently report missing-data rates or handling procedures, raising concerns about potential bias from complete-case analysis or imputation methods.

## 7 Challenges and limitations

### 7.1 Data-Level challenges

GC labels depend on postoperative pathology: Definitive diagnosis of GC requires postoperative pathological examination, meaning that the training labels for ML models are derived exclusively from patients who underwent surgery; unrecognized GC may exist among patients who improved with conservative management, introducing selection bias.

Class imbalance: The incidence of GC among acute cholecystitis cases ranges from approximately 2% to 30%—a relatively low prevalence. Although oversampling methods such as SMOTE are widely employed to address imbalanced data, Ma et al. (17) found that these methods paradoxically exacerbated overfitting in GC prediction. To date, no generally accepted optimal solution exists for effectively handling class imbalance without introducing synthetic data.

Multicenter data heterogeneity: Variations in laboratory testing methodologies, imaging equipment parameters, and clinical practices across centers may lead to performance degradation when models are deployed in new environments. Establishing unified data acquisition standards and preprocessing pipelines is critical to addressing this challenge.

### 7.2 Model-Level challenges

Overfitting risk: Training sample sizes for GC prediction models are relatively limited, whereas deep learning models may involve millions of parameters, rendering overfitting risk non-trivial. Ma et al. (17) mitigated this issue through temporal test set validation and variable reduction strategies; however, more rigorous cross-validation and independent geographical validation remain necessary.

The “black-box” problem and limitations of XAI: Although methods such as SHAP provide model explanations, these explanations may themselves be misleading—correlation does not imply causation, and the “important features” revealed by SHAP may not necessarily reflect true pathophysiological mechanisms. SHAP explanations are also model-specific and can be unstable, with small changes in data or model parameters potentially leading to different feature rankings. Furthermore, current XAI methods are primarily suited for tree-based models on structured data; explanations for complex deep learning architectures remain inadequate.

Lack of calibration and clinical utility reporting: None of the included studies reported calibration curves, decision curve analysis, or net benefit analyses. Model performance was reported almost exclusively in terms of AUC (discrimination), which is insufficient for assessing clinical usefulness. Calibration assesses whether predicted probabilities match observed outcomes, while decision curve analysis quantifies net clinical benefit across threshold probabilities. Future studies must report these metrics to enable meaningful clinical interpretation.

Lack of prospective validation: All published GC-AI models are retrospective studies, lacking prospective validation. This constitutes the greatest barrier to the translation of AI from research into clinical practice. Beyond randomized controlled trials, other study designs may also be appropriate, including prospective silent validation, implementation studies, cluster-randomized trials, and stepped-wedge designs.

### 7.3 Clinical translation challenges

Clinician trust and adoption rates: The clinical adoption of AI prediction models depends on clinician trust. Multiple survey studies have demonstrated that surgeons' acceptance of AI-assisted decision-making correlates positively with model interpretability (43, 44). XAI methods such as SHAP may help build trust by providing individualized explanations; however, whether clinicians truly benefit from these explanations during real-world decision-making requires empirical evaluation.

Legal and ethical boundaries: When AI predictions conflict with clinical judgment, questions of legal liability and decision-making authority arise. These issues are particularly challenging in the context of emergency surgical decision-making—delays in GC management can be fatal, yet AI-driven over-intervention may also expose patients to unnecessary surgical risk.

Accessibility across different tiers of hospitals: Current GC-AI research originates almost exclusively from large tertiary medical centers, and model performance in primary care settings remains uncertain. Given that early identification of GC is most urgently needed precisely at the primary care level, adapting AI tools to resource-limited environments constitutes a critical challenge for clinical translation.

### 7.4 Implementation barriers

Beyond the technical and methodological challenges described above, several practical barriers must be addressed before AI-based GC prediction can be implemented in routine clinical practice:

Regulatory approval: AI-based medical devices require regulatory clearance (e.g., FDA, NMPA, CE marking), which demands evidence of safety, effectiveness, and clinical utility. Current GC-AI studies, being retrospective and lacking prospective validation, would not meet regulatory requirements for clinical deployment.

Integration into hospital workflows: AI models must be integrated into existing clinical workflows, including emergency department triage, surgical consultation, and preoperative assessment. This requires interoperability with electronic health record (EHR) systems, real-time data acquisition, and minimal disruption to clinical processes.

Economic cost: The development, deployment, and maintenance of AI systems involve costs related to computational infrastructure, software licensing, data storage, model updating, and personnel training. Cost-effectiveness analyses are needed to justify the investment relative to existing clinical practices.

Model maintenance and updating: AI models may experience performance degradation over time due to changes in patient populations, clinical practices, or data distributions (model drift). Continuous monitoring, periodic retraining, and quality assurance protocols are essential but add to the implementation burden.

### 7.5 Dataset diversity and generalizability

Most included studies originate from relatively limited geographic regions, particularly China. This raises important concerns regarding model generalizability:

Ethnic and demographic differences: The prevalence of GC risk factors (e.g., diabetes prevalence, cardiovascular disease patterns, gallstone disease characteristics) may vary across ethnic groups and geographic regions. Models trained on predominantly Chinese populations may not generalize to other demographic groups.

Healthcare system variability: Differences in healthcare access, referral patterns, timing of surgical intervention, and perioperative management across healthcare systems may affect both the prevalence and outcomes of GC, potentially limiting model transferability.

Imaging protocol differences: Variations in CT scanner models, acquisition parameters, contrast administration protocols, and ultrasound equipment across institutions and countries may affect the performance of imaging-based AI models. Standardization of imaging protocols is needed before multicenter or international deployment.

Laboratory standardization: Differences in laboratory assay methods, reference ranges, and units of measurement across institutions may introduce variability in model inputs. Harmonization of laboratory data is essential for structured-data ML models.

## 8 Future directions

Based on the current evidence and identified gaps, we propose the following prioritized research directions, distinguishing near-term achievable goals from longer-term speculative possibilities.

### 8.1 Near-term priorities (achievable within 2–3 years)

Prospective external validation: The most immediate priority is prospective, multicenter validation of existing GC-AI models [Ma et al. (17), Hu et al. (18), Guo et al. (19) at independent institutions, particularly outside China, to assess geographical generalizability. Prospective silent validation—where model predictions are generated but not disclosed to clinicians—can evaluate real-world performance without affecting clinical decisions.

Calibration and clinical utility assessment: Future studies must report calibration curves, decision curve analysis, positive and negative predictive values, and net benefit analyses. These metrics are essential for determining whether model predictions translate into clinically meaningful improvements in decision-making.

Head-to-head algorithm comparison: Future studies should conduct direct comparisons of different algorithms (e.g., XGBoost vs. Random Forest vs. deep learning) under unified variable sets, standardized preprocessing, and shared validation cohorts, rather than relying on cross-study AUC comparisons.

Critical appraisal and reporting standardization: Future GC-AI studies should adhere to TRIPOD + AI and PROBAST + AI reporting guidelines, including transparent description of data splitting (patient-level vs. image-level), missing-data handling, and feature-selection procedures to prevent information leakage.

### 8.2 Intermediate-term goals (3–5 years)

Implementation studies: Beyond RCTs, implementation research designs—including prospective silent validation, cluster-randomized trials, and stepped-wedge designs—may be appropriate for evaluating the clinical impact of GC-AI tools in real-world settings. These studies should assess not only diagnostic performance but also clinical outcomes (complication rates, time to surgery, length of stay, healthcare costs).

Federated learning for multicenter collaboration: Federated learning frameworks may enable collaborative model training across institutions while preserving data privacy, potentially improving model generalizability and fairness. However, technical challenges related to data heterogeneity, communication overhead, and model convergence need to be addressed.

Integration with clinical workflows: Developing interoperable AI tools that integrate with EHR systems and hospital information systems is essential for clinical adoption. This requires addressing regulatory approval, standardization of input data, and real-time prediction capabilities.

### 8.3 Longer-term and speculative directions

The following directions are more speculative and should be clearly identified as future research hypotheses rather than near-term expectations:

Multimodal large models: Combining clinical text, laboratory parameters, imaging features, and surgical video within a unified predictive framework is a promising but largely unexplored direction. No GC-specific multimodal model has been published to date.

Edge computing deployment: Deploying lightweight GC prediction models on local devices in primary care settings may address computational resource limitations, though this requires model compression and optimization.

Digital twins: Constructing patient-specific gallbladder digital twin models for simulating disease progression under different interventions remains a forward-looking vision with no published GC-specific studies.

Temporal dynamic modeling: Models that track the temporal evolution of inflammatory markers (e.g., WBC, CRP trajectories) may provide earlier warnings than static models, but no such model has been developed for GC.

### 8.4 Research framework for risk stratification

We propose a conceptual research framework for AI-assisted GC risk stratification. This framework is presented exclusively as a hypothetical research agenda and does not constitute a clinical standard. Any risk thresholds must be derived and validated through prospective studies before clinical application. Management of acute cholecystitis depends on disease severity, physiological status, comorbidities, local expertise, and intraoperative findings—not solely on predicted GC probability. Similarly, conversion to open surgery, subtotal cholecystectomy, and other bailout procedures are primarily intraoperative safety decisions. The proposed framework envisions: (1) low-risk patients may be candidates for observation or elective surgery; (2) intermediate-risk patients may benefit from contrast-enhanced CT evaluation and multidisciplinary discussion; and (3) high-risk patients may warrant early surgical intervention. However, the specific probability thresholds, sensitivity, calibration, and net benefit of such a pathway remain to be determined through prospective studies. It must not be implied that patients considered at low GC risk should routinely receive conservative treatment or elective surgery without appropriate clinical assessment.

### 8.5 Guideline integration

Incorporating AI prediction tools into international standards such as the Tokyo Guidelines represents a long-term goal. Future guideline revisions may consider recommendations for AI-assisted assessment, but only after adequate prospective validation and clinical impact studies have been completed. Any guideline integration must specify the applicable scenarios, limitations, and human-AI collaborative decision-making workflows for AI tools.

## 9 Conclusions

This scoping review examined the application of AI technologies in GC risk factor identification and preoperative prediction. A comparison of traditional scoring systems and AI models is provided in Table 3. Three studies directly addressed AI-based GC prediction, all employing retrospective designs. Machine learning models using structured clinical data achieved temporal or external validation AUCs of 0.818–0.944, while deep learning models integrating non-contrast and contrast-enhanced CT achieved independent validation AUCs of 0.879 and 0.887 (training-set AUC 0.965). Explainable AI methods identified model-associated predictors, including hypokalemia/hyponatremia, though these findings require pathophysiological validation and should not be interpreted as causal risk factors.

**Table 3:** Comparison of traditional scoring systems and AI models for GC prediction.

Feature	Traditional scores (Wu, Yacoub, Bouassida)	ML models (Ma, Hu)	DL models (Guo)	Advantages of AI	Limitations of AI
Algorithm	Logistic regression	XGBoost, Random Forest	seResNet-50 (SSL)	Non-linear relationships; high-dimensional data	Black-box; computational complexity
Input data	Clinical variables (4–6 factors)	Structured clinical data (7–26 variables)	CT images (non-contrast + contrast)	Integrates imaging; more variables	Requires standardized data acquisition
AUC (best reported)	0.75–0.83 (development)	0.818–0.944 (validation)	0.879–0.887 (validation)	Higher discrimination (preliminary)	Not directly comparable across studies
Validation	Mostly development cohort only	Temporal/multicenter external	Independent multicenter	Some external validation available	No prospective validation
Calibration	Not reported	Not reported	Not reported	—	Unknown clinical utility
Decision curve analysis	Not reported	Not reported	Not reported	—	Unknown net benefit
Explainability	Transparent (coefficients)	SHAP feature importance	Limited (no SHAP for DL)	Individualized explanations (ML)	SHAP does not imply causality; DL explanations limited
Clinical interpretability	High (simple scores)	Moderate (SHAP)	Low (black-box)	Individualized risk explanation	Surgeons may not trust explanations
External validation	Limited or absent	Preliminary	Preliminary	Some multicenter evidence	Generalizability uncertain
Prospective studies	None	None	None	—	No evidence of clinical impact
Implementation	Simple (pen and paper)	Online app available	Requires CT infrastructure	Real-time prediction possible	EHR integration; regulatory approval needed
Reproducibility	High (simple formulas)	Low (code not available)	Low (code not available)	—	Independent verification not possible
Cost	Minimal	Low (structured data)	High (CT, computational)	—	Infrastructure and maintenance costs

Direct head-to-head comparisons between traditional scores and AI models have not been performed under unified conditions. AUC values are not directly comparable across studies due to differences in population, predictors, outcome definition, and validation design. GC, gangrenous cholecystitis; ML, machine learning; DL, deep learning; SSL, self-supervised learning; SHAP, SHapley Additive exPlanations; EHR, electronic health record.

However, the evidence base remains insufficient for routine clinical decision-making. Key limitations include: (1) methodological heterogeneity across studies; (2) limited external and prospective validation; (3) absence of calibration, decision curve analysis, and clinical utility metrics; (4) retrospective, surgery-only selection bias; (5) small event numbers and class imbalance; and (6) lack of reproducibility (code and model availability). Statements that specific algorithms “substantially outperform” traditional scores are not supported by direct head-to-head comparisons and should be avoided.

The most immediate research priorities are prospective multicenter validation, calibration and clinical utility assessment, and implementation research. More speculative directions—including multimodal large models, federated learning, edge computing, and digital twins—remain largely hypothetical in the GC context. Until prospective studies demonstrate improved clinical outcomes with AI-assisted decision-making, GC-AI tools should be considered research tools rather than clinical decision-making instruments.
